# Risk factors associated with underweight in children aged one to two years: a longitudinal study

**DOI:** 10.1186/s12889-024-19147-9

**Published:** 2024-07-15

**Authors:** Sara Manoochehri, Javad Faradmal, Jalal Poorolajal, Fatemeh Torkaman Asadi, Ali Reza Soltanian

**Affiliations:** 1 Department of Biostatistics, Student Research Committee, PhD Candidate of Biostatistics, Hamadan University of Medical Sciences, Hamadan, Iran; 2 Modeling of Noncommunicable Diseases Research Center, Hamadan University of Medical Sciences, Shahid Fahmideh Boulevard, Hamadan, Iran; 3 Research Center for Health Sciences and Department of Epidemiology, School of Public Health, Hamadan University of Medical Sciences, Hamadan, Iran; 4 Department of Infectious Disease, School of Medicine, Hamadan University of Medical Sciences, Hamadan, Islamic Republic of Iran

**Keywords:** Multilevel analysis, Failure to thrive, Pediatrics, Growth disorders, Infant, Low birth weight

## Abstract

**Background:**

Underweight is a prevalent health issue in children. This study aimed to identify factors associated with underweight in children aged 1–2 years in Hamadan city. Unlike the studies conducted in this field, which are cross-sectional and do not provide information on the effect of age changes on underweight, our longitudinal approach provides insights into weight changes over time. On the other hand, this study focuses on the high-risk age group of 1 to 2 years, which has only been addressed in a few studies.

**Methods:**

In this longitudinal study, 414 mothers with 1 to 2 year-old children referred to the health centers of Hamadan city, whose information is in the SIB system, a comprehensive electronic system, were examined to identify factors related to underweight. The response variable was weight-for-age criteria classified into three categories: underweight, normal weight, and overweight. A two-level longitudinal ordinal model was used to determine the factors associated with underweight.

**Results:**

Of the children studied, 201 (48.6%) were girls and 213 (51.4%) were boys. Significant risk factors for underweight included low maternal education (AOR = 3.56, 95% CI: 1.10–11.47), maternal unemployment (AOR = 3.38, 95% CI: 1.05–10.91), maternal height (AOR = 0.85, 95% CI: 0.79–0.92), lack of health insurance (AOR = 2.85, 95% CI: 1.04–7.84), gestational age less than 24 years (AOR = 3.17, 95% CI: 16.28–0.97), child age 12–15 months (AOR = 2.27, 95% CI: 1.37–3.74), and child's birth weight (AOR = 0.63, 95% CI: 0.70–0.58).

**Conclusion:**

Based on the results of the present study, it seems that the possibility of being underweight among children is more related to the characteristics of mothers; therefore, taking care of mothers can control some of the weight loss of children.

## Introduction

Underweight is a key indicator of child malnutrition, defined as weight for age less than minus two standard deviations (-2SD) from the reference population median[1]. Maintaining and improving the health of children, one of the most vulnerable populations, is a priority for any society to ensure the future health of society [2]. Monitoring growth patterns of children is essential for daily health care [3]. In developing countries such as Iran, the prevalence of growth disorders and underweight among children remains a major concern, which requires a multidimensional investigation of economic, cultural, and social dimensions [4].

Global statistics highlight the prevalence of underweight among children, particularly in developing regions. In 2018, the prevalence of underweight in Asia was 16.6% [5]. In Iran, the prevalence among children under 5 years of age was 11% in 2018 [6]. Regional disparities within Iran show different rates: Hamadan City recorded 5% among children aged 2–5 years in 2019, Zahedan reported 7.6% among children aged 0–59 months in 2022, Fars Province documented 9.66% among children under 6 years in 2014, and Chadgan in Isfahan Province had 34.5% among children under 5 years in 2011. In contrast, West Azerbaijan Province had a lower prevalence of 4.3% [7–12].

The impact of childhood underweight goes beyond statistics, with an estimated 2.6 million child deaths annually attributed to various forms of malnutrition, a significant proportion of which is underweight [13]. It leads to poor mental health, developmental delays, cognitive impairment, and affects social skills and future opportunities, hindering economic and social advancement [14]. Addressing underweight as a multifaceted problem requires an understanding of economic, social, demographic, health and environmental factors [15].

In contrast to traditional cross-sectional studies, our longitudinal study measures children's weight over time, providing insight into trends in weight change. On the other hand, most studies in this area have been conducted in children under 5 years of age, but this study focuses on children 1–2 years of age, a critical age range for energy-intensive activities such as walking [16]. Therefore, our aim is to fill a significant gap in the existing literature on growth and weight gain trends in this age group.

Understanding the determinants of underweight will help physicians and health care providers plan resources and target special attention to high-risk children. Conducting studies in this area will help health policymakers reduce the risk of underweight through intervention programs and preventive measures.

## Materials and methods

### Study design

This longitudinal study used registered data from mothers and their children aged 1–2 years who visited health centers in the city of Hamadan. Data collection was done through the Integrated Health System (SIB). SIB is a national electronic health record system in Iran which continuously and electronically records include various health indicators and clinical information[8], ensuring comprehensive and reliable data for our study.

### Setting

Four out of 24 centers in Hamadan (a city in western Iran) were selected by simple random sampling. Information from all mothers and children aged 1–2 years who visited these centers during 2022–2023 was reviewed.

### Participants

The study included 414 mother–child pairs with visit data recorded at 12, 15, 18, and 24 months, for a total of 1008 data points. Children with genetic diseases, congenital anomalies associated with developmental disorders, or incomplete medical records were excluded.

### Response Variable

Many studies, including the present one, have used the weight-for-age (WAZ) criterion to check for underweight. According to standard curves, if the WAZ is -3z ≤ WAZ < -2z, the child is underweight; -2z ≤ WAZ ≤  + 1z indicates normal weight; and WAZ >  + 1z indicates overweight[11].

### Predictive variables

Variables related to the child: sex, age of the child (months), birth weight (gr), type of feeding (breast milk/ formula), history of hospitalization (yes/no), premature baby: A baby born before the thirty-seventh week[9] (yes/no), having two or more twins (yes/no), health insurance status (yes/no), birth rank of the child (rank one/two and above), distance from birth of previous child (only child, less than two years, more than two years), Growth monitoring age (12–15 months, 15–18 months, 18–24 months).

Variables related to maternal characteristics: maternal age (years), gestational age (years), maternal education level (less than diploma/diploma/academic), maternal employment status (unemployed/employed), pre-pregnancy BMI (kg/${m}^{2}$), pre-pregnancy weight (kg), height (cm), history of abortion (yes/no), planned pregnancy (wanted/unwanted), type of delivery (cesarean/natural), place of residence (urban/suburban: Tissues that are mainly rural migrants and they have urban poor schools) [10], health status of the mother (healthy, thyroid disease, diabetes, thalassemia, pre-eclampsia, other). It should be noted that since the information on the demographic characteristics of more than 20% of the fathers was not complete, these variables were not studied.

### Data sources/measurement

All weight measurements were performed by midwives using a digital smart scale with an accuracy of 100 gr. In this study, the standard growth curve of the US National Center for Health Statistics was used to monitor the growth status of the children.

### Statistical methods and software

2.7.1. Multilevel ordinal logistic regression model.

**Fig. 1:**
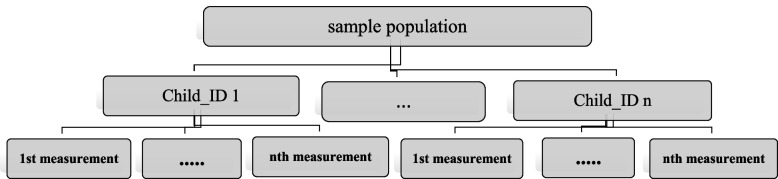
illustrates the schematic of the 'Multilevel Diagram' for a two-level model, where repeated observations (level 1) are nested within individuals (level 2) in a study of children aged 1–2 years, incorporating repeated measures over time

In this longitudinal study, a two-level model was used to model the factors associated with childhood underweight. In two-level models, repeated observations (level 1) are nested within individuals (level 2). In the present study, the repeated measures include the weight of children aged 1 to 2 years measured at 12 to 24 months (level 1), and these measurements were nested within children (level 2). This hierarchical structure is shown in Fig. 1. The two-level ordinal logistic model with width from random origin is shown as follows:

1 $$logit\ \left[P\left({Y}_{i}\le j\right)\right]={\alpha }_{j}+{X}^{\prime}\beta +{u}_{i}\ {\mathrm {j=1,2...J;}}\ {\alpha }_{J}=0$$

In this expression, ${\alpha }_{j}$, related to J-1intercept,for the J ordinal model, X is the design matrix related to the fixed effects, β is the vector of regression coefficients or the effect of the studied variables, ${u}_{i}$ is the random intercept corresponding to the i^th^ child, which is assumed to have a normal distribution with zero mean. In the present study, ${Y}_{i}$ represents weight groups for age, considered as underweight, normal and overweight (J = 3). Model 1 is called a proportional odds model because the effect of each factor (β) is the same across outcome categories [12].

### Software

The SPSS version 24 software was used to describe the data, and the clmm2 function available in the ordinal package of the R4.0.3 software was used to univariate of the relationship between the factors and the response variable, as well as to fitting the two-level ordinal logistic regression model. The significance level in this study was considered to be less than 5%.

## Results

### Characteristics for children

In this study, which was conducted on 414 pairs of mothers and children aged 12 to 24 months in Hamadan city, the mean age of 414 children was 19.62 ± 2.81 months and their mean birth weight was 3096.51 ± 500.14 gr. the results showed that 48.6% (201 people) of the children were girls and 51.4% (213 people) were boys. Almost three quarters of the children (72.2%) were breastfed. The proportion of children born preterm was 14.7%. Almost two-thirds (66.8%) of the children in this study were covered by insurance. Of the 414 children studied, only 14 children (3.4%) were twins. In this study, 48.1% of the children were the first child and 51.9% were the second child or older. About half of the children (47.8%) lived in the suburbs of Hamadan.

### Maternal characteristics

The mean age of the 414 mothers was 32.24 ± 5.54 years, the mean gestational age was 30.79 ± 5.47 years, the mean pre-pregnancy weight was 10.42 ± 67.61 kg, and their mean height was 161.58 ± 5.90 cm. Based on the results, most of the mothers (64.5%) had delivered by cesarean section. Regarding unwanted pregnancy, 80 mothers (19.3%) had unwanted pregnancy. For 28.7% of mothers, a history of abortion was reported at least once. About 24% of mothers have less than diploma education, 30.9% have diploma and 45.2% have academic education. Among them, about a quarter (25.1%) of the mothers work outside the home. 34.3% had a history of various underlying diseases including thyroid disease, pre-eclampsia, diabetes, thalassemia and others.

The results presented in Table 1 are based on information from 1008 visits by 414 children, so a child may have visited the health center several times during a year (from 12 to 24 months). According to the results of this table, 498 children were referred in the first quarter, 216 children in the second quarter, and 294 children in the last visit.

**Table 1:** Weight gain status across time by group

		**Time point of visit**		
Weight gain	**12month—15month**	**15month -18month**	**18month -24month**	**Total**
Under weight	106	36	50	192
Normal weight	302	135	187	624
Over weight	90	45	57	192
Total	498	216	294	1008

**Fig. 2:**
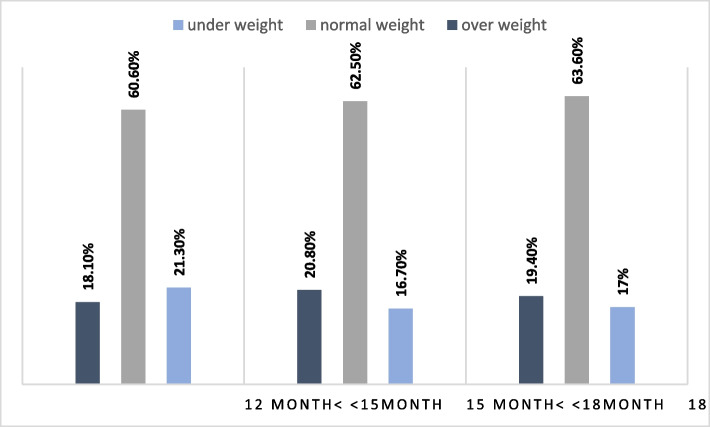
Weight gain status a cross time by group response

The results presented in Fig. 2 show that at the first visit, 21.3% of the children were underweight, which decreased to 16.7% at the second visit and to 17% at the last visit.

Table 2 presents the information of 1008 observations by different categories of the response variable WAZ. This table also shows the analysis of the relationship between variables related to mother and child with the response variable.

**Table 2:** Socio-Demographic and health Characteristics children 1–2 years and of mothers in Hamadan (n = 1008)

**Quantitative features**		**Under weight (Mean ± SD)**	**Normal weight (Mean ± SD)**	**Over weight (Mean ± SD)**	**Total** **(Mean ± SD)**	***p*-value**
**Birth weight (gr)**		2728.4 ± 591.2	3113.7 ± 430.9	3385.4 ± 393.8	3092.08 ± 502.5	< 0.001
**Age child(month)**		20.04 ± 2.94	19.85 ± 2.78	19.77 ± 2.51	19.87 ± 2.76	0.742
**mother's height(cm)**		159.61 ± 5.61	161.47 ± 5.41	164.26 ± 7.19	161.64 ± 6.005	< 0.001
**Pre-pregnancy weight(kg)**		65.28 ± 10.38	67.44 ± 10.25	70 ± 9.99	67.52 ± 10.32	0.003
**Gestational age(year)**		30.93 ± 6.1	30.61 ± 5.20	31.42 ± 5.21	30.82 ± 5.41	0.735
**Mother's age(year)**		33.41 ± 6.28	33.07 ± 5.24	33.93 ± 5.51	33.30 ± 5.51	0.693
**Qualitative features**		**Count (%)**				
		**Under weight (*n*** **= 192)**	**Normal weight (*n*** **= 624)**	**Over weight (*n *= 192)**	**Total** **(*n *= 1008)**	***p-*value**
**Sex child**	**Female**	85(17.3)	330(67.3)	75(15.3)	490(48.6)	0.264
**Male**	107(20.7)	294(56.8)	117(22.6)	518(51.4)		
**Nutrition style**	**Breast feeding**	140(19.0)	458(62.2)	138(18.8)	736(73)	0.781
	**Formula feeding**	52(19.1)	166(61.0)	54(19.9)	272(27)	
**History of hospitalization of the child**	**Yes**	72(47.4)	75(49.3)	5(3.3)	152(15.1)	< 0.001
	**No**	120(14.0)	549(64.1)	187(21.8)	856(84.9)	
**Premature infants**	**Yes**	72(47.4)	75(49.3)	5(3.3)	152(15.1)	< 0.001
	**No**	120(14.0)	549(64.1)	187(21.8)	856(84.9)	
**Type of delivery**	**Natural childbirth**	65(18.0)	222(61.5)	74(20.5)	361(35.8)	0.623
	**Cesarean**	127(19.6)	402(62.1)	118(18.2)	647(64.2)	
**Twins**	**Yes**	14(36.8)	23(60.5)	1(2.6)	38(3.8)	0.006
	**No**	178(18.4)	601(62.0)	191(19.0)	970(96.2)	
**Birth order**	**1st**	97(19.6)	302(60.9)	97(19.6)	496(49.2)	0.982
	**2nd or more**	95(18.6)	322(62.9)	95(18.6)	512(50.8)	
**Distance from the previous child(year)**	**0**	97(19.9)	299(61.4)	91(18.7)	487(48.3)	0.736
	** < 2**	12(16.4)	48(65.8)	13(17.8)	73(7.2)	
	** > 2**	83(18.5)	277(61.8)	88(19.6)	448(44.4)	
**Insurance status child**	**Yes**	108(16.1)	429(63.9)	134(20.0)	671(66.6)	0.013
	**No**	84(24.9)	195(57.9)	58(17.2)	337(33.4)	
**Underlying disease mother**	**Yes**	67(19.3)	219(62.9)	62(17.8)	348(34.5)	0.395
	**No**	125(18.9)	405(61.4)	130(19.7)	660(65.5)	
**History of abortion**	**Yes**	54(18.4)	188(64.2)	51(17.4)	293(29.1)	0.513
	**No**	138(19.3)	436(61.0)	141(19.7)	715(70.9)	
**Mother's education**	** < Diploma**	53(21.2)	153(61.2)	44(17.6)	250(24.8)	0.043
	**Diploma**	59(18.5)	193(60.5)	67(21.0)	319(31.6)	
	**Academic**	80(18.2)	278(63.3)	81(18.5)	439(43.6)	
**Mother occupation**	**Unemployed**	162(21.2)	460(60.3)	141(18.5)	763(75.7)	0.032
	**Employed**	30(12.2)	164(66.9)	51(20.8)	245(24.3)	
**Gestational Age(year)**	** < 24**	36(25.4)	90(63.4)	16(11.3)	142(14.1)	0.007
	** > 24**	156(18.0)	534(61.7)	176(20.3)	866(85.9)	
**Time visit (months)**	**12–15**	106(21.3)	302(60.6)	90(18.1)	498(49.4)	0.003
	**15–18**	36(16.7)	135(62.5)	45(20.8)	216(21.4)	
	**18–24**	50(17.0)	187(63.6)	57(19.4)	294(29.2)	
**Residence**	**Urban**	99(19.0)	323(61.9)	100(19.2)	522(51.8)	0.431
	**Surban**	93(19.1)	301(61.9)	92(18.9)	486(48.2)	

Among 1008 measurements, 19.1% (192 observations) are underweight, 61.9% (624 observations) are normal weight, and 19% (192 observations) are overweight. The results in Table 2 show a significant relationship between different categories of child weight (underweight, normal and overweight) and factors such as mother's age during pregnancy (< 24 years, > 24 yearsyears), pre-pregnancy weight, mother's education, mother's occupation, insurance status, child's birth weight, prematurity, having twins, time of child's visit to the health center (significance level was considered 5%).

Among the 1008 observations examined based on the weight response variable, the mean birth weight of underweight children was 2861.92 ± 537.07 gr, normal weight children were 3149.57 ± 6.02 gr, and overweight children were 3293.20 ± 429.04 gr, which were significantly different from each other (*p*-value < 0.001). The mean height and pre-pregnancy weight of mothers of underweight children were significantly lower than the mean height (p-value < 0.001) and pre-pregnancy weight (*p*-value = 0.003) of mothers of overweight and normal weight children.

Results showed higher rates of underweight in preterm infants compared to term infants (47% vs. 14%, *p* < 0.001). The prevalence of underweight was also higher in twins compared to singletons (36% vs. 18%, *p* = 0.006) and in uninsured children compared to insured children (24.9% vs. 16.1%, *p* = 0.013). The prevalence of underweight was highest in children aged 12–15 months compared to older age groups (*p* = 0.003). In addition, the prevalence of underweight was higher among children with mothers with less than a high school education (*p *= 0.043), unemployed mothers (*p* = 0.032), and mothers younger than 24 years of age at pregnancy (*p* = 0.007).

After fitting the generalized two-level model, the results of which are presented in Table 3, the significant variables related to the child: the child's birth weight, the child's growth monitoring age, and the significant variables related to the mother: age at conception (less than 24 years, more than 24 years), education, occupation, height, and insurance status.

**Table 3:** Estimates and the 95% confidence intervals for parameters proportional odds model

	Adjusted			Unadjusted		
**Variable**	Estimated(SD)	OR (95% CI)	*P-*value	Estimated(SD)	OR(95% CI)	*P*-value
Birth weight (gr)	-0.45 (0.05)	0.63 (0.58,0.70)	< **0.001**	-0.44 (0.05)	0.64 (0.58,0.71)	** < 0.001**
**Growth monitoring (Ref = 18 to 24 months old)**	-	-	-	-	-	-
12 to 15 months old vs Ref	0.82(0.26)	2.27 (1.37,3.74)	${0.001}^{*}$	0.75(0.25)	2.11(1.29,3.45)	${0.003}^{*}$
15 to 18 months old vs Ref	0.30(0.34)	1.34 (0.69,4.39)	0.362	0.44(0.29)	1.55(0.88,2.71)	0.126
**Mother's education(Ref = Academic)**	-	-	-	-	-	-
Diploma vs Ref. >	1.27(0.60)	3.56 (1.10,11.47)	${0.031}^{*}$	1.25(0.60)	3.49(1.08,11.24)	${0.012}^{*}$
Diploma vs Ref	0.85(0.66)	2.33 (0.64,8.49)	0.194	0.81(0.66)	2.24(0.61,8.1)	0.156
**Mother occupation (Ref = Employed)**	-	-	-	-	-	-
Un-employed vs Ref	1.22 (0.60)	3.38 (1.05,10.91)	${0.043}^{*}$	1.20 (0.57)	3.32(1.09,10.07)	${0.033}^{*}$
Insurance (Ref = Yes)	-	-	-	-	-	-
No vs Ref	1.05(0.52)	2.85 (1.04,7.84)	${0.041}^{*}$	1.36(0.57)	3.93(1.28,11.82)	${0.011}^{*}$
**Gestational Age(Ref > 24 year)**	-	-	-	-	-	-
< 24 year vs Ref	1.38(0.72)	3.17 (0.97,16.28)	${0.035}^{*}$	1.20(0.69)	3.32(0.86,12.80)	${0.040}^{*}$
Mother height	-0.16(0.04)	0.85 (0.79,0.92)	$<{0.001}^{*}$	-0.21(0.04)	0.81(0.75,0.87)	$<{0.001}^{*}$
Pre -pregnancy weight	0.029 (0.02)-	0.97 (0.93,1.01)	0.28	-0.066(0.02)	0.93(0.50,1.03)	0.183
**Twines (Ref = No)**	-	-	-	-	-	-
Yes vs Ref	1.20(0.81)	3.32 (0.68,16.44)	0.38	1.16(0.78)	3.18(0.69,14.58)	0.311

^*^ significant test in level 0.05

A child's birth weight has a significant effect on the odds of being underweight, so that for every 100 gr increase in a child's birth weight, the odds of being underweight compared to normal and overweight decreases by 0.63 (Adjusted Odds Ratio(AOR) = 0.63, 95% CI: 0.58–0.70, P-value < 0.001). Also, the odds of being underweight compared to normal weight and overweight in children aged 12–15 months are 2.27 times higher than in children aged 18–24 months (AOR = 2.27, 95% CI: 1.37–3.74, P-value = 0.001).

Among children whose mothers have less than a diploma education, the odds of being underweight compared to normal weight and overweight are significantly higher than among children whose mothers have an academic education (AOR = 3.56, 95% CI: 1.10–11.47, *P*-value = 0.031). Also, the odds of being underweight in children whose mothers are not employed are 3.38 times higher than in children whose mothers are employed (AOR = 3.38, 95% CI: 1.05–10.91, P-value = 0.043).In children whose mother and child had no health insurance, the odds of being underweight compared to normal and overweight are 2.85 times higher than in children whose mother and child had insurance (AOR = 2.85, 95% CI: 1.04—7.84, *P*-value = 0.041). Mothers whose gestational age is less than 24 years are more likely to have underweight children (AOR = 3.17, 95% CI: 0.97–16.28, *P*-value = 0.035). On the other hand, a 1 cm increase in maternal height reduces the odds of underweight by 0.85 (AOR = 0.85, 95% CI: 0.92–0.79, *P*-value < 0.001).

## Discussion

The present study was conducted with the aim of investigating the factors affecting the incidence of underweight among children in Hamadan city, using a two-level ordinal regression model. Based on the results obtained from this study, the variables of low education of the mother and her lack of employment, young age of mother during pregnancy, short stature of mother, mother and child not using health insurance services, (Low Birth Weight) LBW and the time of child's visit to the health center to monitor growth, they were one of the factors affecting the incidence of underweight in children aged 1 to 2 years.

In our study, low maternal education played a significant role in the incidence of underweight children, consistent with the findings of Khan's study in Pakistan (OR = 2.55) [13]. Similarly, Prasad's findings in India showed that a total of 60% of short children and 56.6% of underweight children had illiterate mothers [14]. Popat in India [15], Abebe in Ethiopia [16], and Acquah in Ghana [6] have also shown that low maternal education effectively increases underweight. Mothers' lack of knowledge about optimal child care practices, how to navigate the health system, when to start and how to prepare complementary foods, and how to properly use supplements can lead to underweight and stunting after 6 months of age [2].

According to the results of this study, children whose mothers do not work are more likely to be underweight than children whose mothers work. Anil Sigdel in Nepal (OR = 5.13) [17], Nankinga in Uganda [18], and Fitzsimons in England [19] also found similar results to our study. However, Prasad in India[14] and Ketema in Ethiopia, who pointed out that working mothers usually spend less time on their children's health [20], found contradictory results to our study. The reason for this discrepancy may be the different government policies to support women after childbirth. In Iran, working mothers can take up to 9 months of paid leave and can go home for a few hours to breastfeed until the child is 2 years old.

The association between LBW and weight gain observed in this study is consistent with the findings of the Aboagye studies in sub-Saharan Africa (OR = 1.82) [21], Arup Jana in India (OR = 1.76) [22], Khan in Pakistan (OR = 1.67) [13], and Asmare in The Gambia [23]. This association may be because LBW children are prone to infections such as diarrhea, prolonged hospitalization, inability to breastfeed, and loss of appetite, leading to poor physical growth and development [24].

Similar to our findings, Wemakor in Ghana [25], Nguyen in Bangladesh [26], and Sadaf Khan in Pakistan [13] concluded that mothers who conceive at a young age are more likely to have children with low birth weight. Because young mothers need a lot of nutrients for full growth, the mother's body competes with the growing fetus for nutrients during pregnancy, resulting in the child's lack of growth and development and low weight. On the other hand, the lack of psychological preparation of teenage mothers for breastfeeding and their lack of experience or in caring for a baby after delivery may also be other factors [2].

According to the results of the present study, consistent with findings from Sadaf Khan (OR = 2.31) [13], Porwal (OR = 1.64) [27], and Zhihui Li (OR = 3.5) [28], children born to short mothers are more likely to be underweight. The smaller physical structure of the uterus and pelvis in short mothers results in inadequate delivery of nutrients to the fetus, leading to preterm birth or low birth weight [29]. On the other hand, short maternal height usually results in difficult deliveries and cesarean sections, which interfere with breastfeeding in the early days and inadequate colostrum intake, resulting in LBW[30].

In our study, maternal pre-pregnancy weight was not found to be significant. However, a study by Chao Li in China (OR = 2.02) [31] and Porwal (OR = 2.14) in India [32] found that low maternal pre-pregnancy weight increased the risk of low birth weight. The reason for this discrepancy could be that in our study, the occurrence of mothers with low weight was observed in only 17 instances.

Based on the results of the present study, there was no significant association between having twins and the chance of children being underweight. However, the results of Birhan study in Ethiopia (OR = 2.01) [6], Debeko study in Ethiopia (OR = 2.43) [32] and Asmare study in Gambia (OR = 1.15) [23] show the relationship between these two variables. The reason for this discrepancy may be due to the low frequency of twins in our study (14 children).

The results of our study showed that the odds of being underweight were higher among children whose mothers and children had no health insurance than among children whose mothers and children had health insurance. These findings align with the study by Kofinti in 35 African countries [33] and Herman in Indonesia (OR = 1.99) [34]. The use of health insurance services increases access to health care for both mother and child.

In line with our study, Adhikari et al. in Nepal also concluded that regular monitoring of child growth reduces the odds of being underweight (OR = 0.35) [35]. Regular monitoring of child growth improves health care and preventive treatment programs through increased awareness and counseling of parents. On the other hand, encouraging mothers and promoting breastfeeding and appropriate complementary feeding improves feeding practices and thus reduces child underweight [36].

## Limitations

Since in the present study the registered information of the studied samples was used in the SIB system and access to the studied samples was not possible, in cases where the information was incomplete, that sample was discarded and this problem caused a large number of samples to be removed from the study. Furthermore, retrieving information from health centers proved time-consuming as their data was not registered in the SIB system. Additionally, because the SIB system's information is predetermined, certain variables related to underweight were not recorded, resulting in a lack of investigation into some aspects of underweight in this study.

One additional limitation of the present study was the exclusion of a certain number of children with genetic diseases or congenital abnormalities related to developmental disorders. However, given that these samples comprised less than 5% of the total, their exclusion did not significantly impact the results.

## Conclusion

Based on the results of this study, factors related to children's underweight are mother's low education and lack of employment, mother's young age during pregnancy, mother's short stature, mother and child not using health insurance services, LBW and the time the child's visit to the health center was for growth monitoring. Based on the results of the present study, it seems that the probability of children being underweight is more related to the characteristics of mothers; therefore, taking care of mothers can control some of the children's weight loss. In this study, the mother's lack of employment has caused a decrease in family income and ultimately affects weight loss. Therefore, sending support packages to low-income families can be effective in reducing the incidence of underweight children. Educating the community, especially woman who become pregnant at a young age increases the necessary information about pregnancy and ultimately reduces the risk of underweight children. Primary health care (PHC) centers can play an important role in this area.

## Data Availability

The data set analyzed during the present study is not available to the public because it belongs to the “SIB” system and has a limited use license, but is available at the reasonable request of the corresponding author.
